# Efficacy of Power Ultrasound-Based Hurdle Technology on the Reduction of Bacterial Pathogens on Fresh Produce

**DOI:** 10.3390/foods12142653

**Published:** 2023-07-10

**Authors:** Xinyi Zhou, Joelle K. Salazar, Megan L. Fay, Wei Zhang

**Affiliations:** 1 Department of Food Science and Nutrition, Institute for Food Safety and Health, Illinois Institute of Technology, Bedford Park, IL 60501, USA; 2 Division of Food Processing Science and Technology, U.S. Food and Drug Administration, Bedford Park, IL 60501, USA; megan.fay@fda.hhs.gov

**Keywords:** power ultrasound, fresh produce, *L. monocytogenes*, *S.* Newport, hurdle technology, chemical sanitizers

## Abstract

Minimally processed produce is frequently contaminated with foodborne bacterial pathogens. Power ultrasound is a non-thermal and cost-effective technology that can be combined with other chemical sanitization methods. This study investigated the reduction of *Listeria monocytogenes* and *Salmonella* Newport on grape tomato, romaine lettuce, and spinach washed with water, chlorine, or peroxyacetic acid alone or in combination with 25 or 40 kHz power ultrasound for 1, 2, or 5 min. Produce items were inoculated with either pathogen at 10 log CFU/g, dried for 2 h, and treated. Combined treatment of ultrasound and sanitizers resulted in 1.44–3.99 log CFU/g reduction of *L. monocytogenes* and 1.35–3.62 log CFU/g reduction of *S.* Newport, with significantly higher reductions observed on grape tomato. Synergistic effects were achieved with the hurdle treatment of power ultrasound coupled with the chemical sanitizers when compared to the single treatments; an additional 0.48–1.40 log CFU/g reduction of *S.* Newport was obtained with the addition of power ultrasound on grape tomato. In general, no significant differences were observed in pathogen reductions between the ultrasound frequencies, the sanitizers, or the treatment lengths. Results from this study suggest that incorporation of power ultrasound into the current washing procedure may be beneficial for the reduction, but not elimination, of bacterial pathogens on certain produce items, including tomatoes.

## 1. Introduction

Fresh fruits and vegetables are part of a healthy diet, provide nutrients such as vitamins, minerals, and fiber, and can lower the risk of diabetes and cardiovascular diseases [1]. According to the International Fresh Produce Association (IFPA), the market value of products in the U.S. in 2021 reached 71.6 billion USD, representing a 14.4% increase compared to the total fresh produce sales in 2019 [2]. New challenges in production, processing, and food safety for the fresh produce industry have emerged as the market continues to expand substantially.

In recent decades, pathogens such as norovirus, Shiga toxin-producing *Escherichia coli*, *Listeria monocytogenes*, and *Salmonella enterica* have been repeatedly linked to foodborne disease outbreaks associated with fresh produce in the U.S. [3,4,5,6]. From 2009 to the first quarter of 2023, *S. enterica* caused 83 multistate outbreaks related to fresh produce, with 6826 illnesses and 18 deaths. *E. coli* was implicated in 38 multistate outbreaks, mostly related to leafy greens, which led to 1252 illnesses and 7 deaths. Eleven multistate listeriosis outbreaks associated with fresh produce caused 259 illnesses and 56 deaths [4]. Between 2009 and 2022, fresh produce outbreaks accounted for over 30% of the total multistate outbreaks [4]. Norovirus was the leading cause of foodborne disease outbreaks related to fresh produce, whereas outbreaks linked to bacterial pathogens resulted in higher fatality rates.

The fresh produce industry often uses cold water supplemented with chlorine-based sanitizers as an attempt to reduce the populations of bacteria on produce surfaces as part of a “minimal processing” of freshly harvested produce items. Other chemical sanitizers, such as peroxyacetic acid (PAA) and acidified sodium chlorite, have also been approved by U.S. FDA and the Environmental Protection Agency (EPA) for produce washing. Generally, the concentrations of chlorine and PAA for produce wash range from 40 to 200 ppm (free chlorine) and 20 to 80 ppm, respectively [7]. However, chlorine or PAA alone is often inadequate to achieve complete pathogen inactivation in fresh produce. Fresh produce contains large amounts of organic compounds, which can reduce the efficacy of chlorine and PAA. Chemical degradation also leads to the inefficacy of chlorine and PAA in reducing the pathogen load. The inadequacy of chemical-based wash practices and the increasing number of fresh produce-related human foodborne outbreaks highlight the need for evaluating and incorporating more effective disinfection technology to assist in inactivating bacterial pathogens in fresh produce.

To preserve the nutrition and freshness of fresh produce, traditional thermal processing is not an option. Alternative methods, such as nonthermal ultrasound, may provide additional microbial inactivation while retaining fresh characteristics. Ultrasound technology is generally classified into two groups of applications based on the frequency used: high-frequency ultrasound and power ultrasound. High-frequency ultrasound uses frequencies ranging between 2 to 20 MHz and is commonly used for disease diagnosis. Power ultrasound uses lower frequencies between 20 to 100 kHz and generates higher energy. This technology is widely used for medical equipment cleaning and decontamination [8]. Power ultrasound inactivates bacteria through the cavitation effect. When acoustic energy exceeds the cavitation threshold in liquid media, microbubbles quickly form, grow, and collapse. The shear forces generated by the collapse of the ultrasound cavities near the surface of bacteria lead to the breakage of cell membranes and leakage of cytoplasm [9,10]. In addition, the shock wave and turbulence of microbubble bursts can loosen up and “shake off” pathogenic bacteria that are attached to those “hard-to-reach” places, such as cavities and grooves on the product surface, making chemical sanitization more effective [11,12,13]. Power ultrasound is a non-thermal, eco-friendly, and cost-effective technology which can be implemented in producing washing lines as a supplemental method to sanitizer washing [14,15].

Several studies have demonstrated the potential of power ultrasound alone or combined with antimicrobial compounds such as chorine, PAA, or organic acids to reduce the bacteria load on the fresh produce surfaces [13,16,17,18,19,20]. However, many of the studies evaluated washing conditions that were not practical for industry use (e.g., long treatment times). There are also wide variations among the collected data in these studies due to the different experimental procedures, ultrasound setups, and treatment conditions, which makes it challenging to compare the effects of the efficacy of ultrasound. More information is needed in the evaluation and validation of optimal washing conditions for the fresh produce industry to adopt power ultrasound technology in practical use.

To evaluate the efficacy of ultrasound as a “hurdle” technology in combination with chemical sanitizers for fresh produce wash, we compared population dynamics of *Salmonella enterica* and *Listeria monocytogenes* on several fresh produce items with distinct surface characteristics using different combinations of ultrasound frequencies and wash times in this study. The results of this study can provide useful assessments on the practicality of implementing power ultrasound in the minimal processing of fresh produce.

## 2. Materials and Methods

### 2.1. Fresh Produce

Spinach, iceberg lettuce, and grape tomatoes were obtained from local grocery stores (Chicago, IL, USA), stored at 4 °C, and used within 4 days. All samples used in the experiment were free of visible surface damage.

### 2.2. Inoculum Preparation

*S. enterica* Newport strain 36796 (CFSAN046260, a tomato outbreak isolate) and *L. monocytogenes* strain LS810 (a cantaloupe outbreak isolate [21]) were obtained from U.S. FDA stock culture collection in Bedford Park, IL. Strain LS810 was resistant to rifampicin at 200 µg/mL via stepwise exposure to increasing concentrations of the antibiotic. Working stocks of both strains were maintained on Tryptic Soy Agar (TSA; Becton, Dickinson and Co., Sparks, MD, USA) or Brain Heart Infusion Agar (BHIA; Becton, Dickinson and Co.) supplemented with rifampicin (BHIA^rif^) for *S.* Newport and *L. monocytogenes*, respectively. Liquid culture was prepared by inoculating one colony in 30 mL of Tryptic Soy Broth (TSB; Becton, Dickinson and Co.) and incubating at 37 °C for 16–18 h. Bacterial cells were harvested by centrifuging at 4000 × *g* and 4 °C for 15 min. The cell pellets were washed twice with 30 mL Butterfield’s Phosphate Broth (BPB) and then re-suspended in 3 mL BPB to obtain ca. 10 log CFU/mL inoculum. The concentration of inoculum was verified by plating serial dilutions of liquid culture onto TSA or BHIA^rif^ accordingly.

### 2.3. Washing Solutions Preparation

Three washing solutions were used: sterile water (W), 40 ppm PAA (P), and 50 ppm Chlorine (C). P solution was prepared by diluting concentrated Tsunami-100 (Ecolab, St. Paul, MN, USA) in sterile water; the final concentration was measured using the peroxyacetic/hyd peroxide test kit (Ecolab) according to the manufacturer’s instructions. C solution was prepared by diluting sodium hypochlorite solution (12% available chlorine, Spectrum Chemical, New Brunswick, NJ, USA) in sterile water; the concentration of free chlorine was measured by using the pocket chlorine meter (Hach, Loveland, CO, USA) before each trial according to the manufacturer’s instructions. The effective concentrations of both P and C solutions were monitored every 2 min throughout the washing treatments.

### 2.4. Fresh Produce Inoculation and Treatment

Twenty-five grams of spinach or iceberg lettuce were weighed on foil pans (20 × 20 cm) and spot-inoculated with 20 spots of 50 µL inoculum each. Grape tomatoes (100 g) were spot inoculated with 1 mL of inoculum (10 µL per spot) in a 240-mL capacity deli container. After inoculation, all samples were air-dried for 2 h at ambient temperature in a biosafety cabinet.

Washing treatments in this study were conducted as described previously [20] with modifications. Briefly, two ultrasonic cleaners equipped with 25 kHz (30.8 W/L, model: XPD360-6L, Sharpertek, Pontiac, MI, USA) or 40 kHz (30 W/L, model: UZK-5.2, Shield Ultrasonic, Calabasas, CA, USA) transducers underneath the water tanks were used. Beakers (2-L capacity) containing 1 L washing solution were placed in the ultrasonic cleaner tank filled with water. Ultrasonicators were run for 5 min to degas the water before each treatment. Samples were transferred into beakers aseptically and treated with the following conditions: W, W with 25 or 40 kHz ultrasound (W25/40U), C, C with 25 or 40 kHz ultrasound (C25/40U), P, and P with 25 or 40 kHz ultrasound (P25/40U). All treatments were conducted for 1, 2, or 5 min. After treatments, samples treated with W and W25/40U were transferred into 1-L capacity stomacher bags immediately. For those treated with C or P, 2 mL of 1 M sodium thiosulfate (Fisher Scientific, Waltham, MA, USA) was added into the beakers and mixed for 1 min for neutralization. Samples were then transferred into stomacher bags. Unwashed inoculated samples were used as negative controls.

### 2.5. Microbiological Analysis

The microbiological analysis for each sample was conducted immediately after the treatment. Twenty-five g of spinach and iceberg lettuce were homogenized with 225 mL BPB for 1 min using a stomacher (paddle lab blender, Neutec Group, Farmingdale, NY, USA). For 100 g tomatoes, 100 mL of BPB was added, and the sample was homogenized by a stomacher for 1 min. For *S.* Newport, serial dilutions of the homogenates were spread plated onto TSA overlayed with Xylose Lysine Deoxycholate agar (XLD, Becton, Dickinson and Co.). For *L. monocytogenes*, serial dilutions of the homogenates were plated onto BHIA^rif^. Agar plates were incubated at 37 °C for 24 h; TSA were incubated for 24 h, while BHIA^rif^ were incubated for 48 h prior to bacterial colony enumeration. Data were expressed as log CFU/g.

### 2.6. Statistical Analysis

Two technical replicates were included for each treatment in each trial, and four trials (biological replicates) were performed on separate days (*n* = 8). Student’s t-test was used to compare population reductions between two pathogens on the same matrix under the same treatment condition (W, W25U, W40U, C, C25U, C40U, P, P25U, or P40U) and time (1, 2, or 5 min). One-way ANOVA with Tukey’s Post-hoc test was used to compare the populations of a single pathogen between (1) different treatment conditions at the same treatment time on the same matrix, (2) different treatment times for the same treatment condition on the same matrix, and (3) different matrices which were treated the same (treatment condition and time). A *p* value less than 0.05 was considered to be significant.

## 3. Results

### 3.1. Sanitizer Concentration Monitoring

Sanitizer concentrations used in the project were kept at 50.59 ± 1.58 ppm and 40.00 ± 5.00 ppm for free chlorine and PAA, respectively, and no significant degradation was observed for both chemicals during treatments due to the small sample size with minimal organic matter.

### 3.2. Population Dynamics of S. enterica Newport on Fresh Produce

Samples inoculated with *S.* Newport were subjected to washing treatments using W, W25U, W40U, C, C25U, C40U, P, P25U, and P40U for 1, 2, or 5 min. Prior to treatment, the *S.* Newport populations inoculated on tomato, spinach, and iceberg lettuce were 7.44 ± 0.26, 8.32 ± 0.17, and 8.56 ± 0.23 log CFU/g samples, respectively.

**Figure 1:**
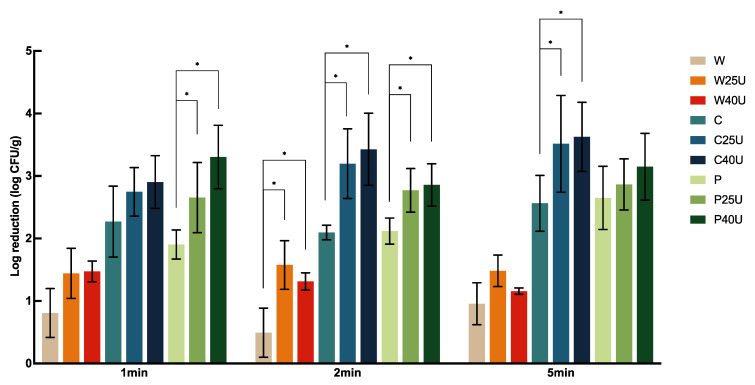
Population dynamics of *Salmonella enterica* Newport 36796 on grape tomato treated with water (W), water with 25/40 kHz ultrasound (W25/40U), chlorine (C), chlorine with 25/40 kHz ultrasound (C25/40U), peroxyacetic acid (P), and peroxyacetic acid with 25/40 kHz ultrasound (P25/40U) for 1, 2, and 5 min. Data are represented as mean and standard deviation (*n* = 8). Selected statistical analysis results on the figure depict significant differences between population reductions on grape tomatoes treated with the same washing solution with and without ultrasound; * indicates significant differences in populations. All other significant differences are indicated in the text.

The log CFU/g reduction of *S.* Newport population on grape tomato under different treatments is shown in Figure 1. Treatment with water (W), 50 ppm chlorine (C), or 40 ppm PAA (P) for 1 min resulted in 0.81 ± 0.39, 2.27 ± 0.57, or 1.90 ± 0.23 log CFU/g reduction, respectively, with significantly greater reduction caused by both C and P compared to W but with no significant difference between the two sanitizers. For tomato samples treated with W, C, and P, no significant difference was observed among the majority of treatment times. One exception was that washing with P for 5 min resulted in significantly greater reductions of 0.75 and 0.53-log CFU/g compared to the 1- and 2-min treatments.

Overall, ultrasound increased the reduction of *S.* Newport on tomato by 0.48–1.4-log CFU/g depending on the washing solution used. A combination of ultrasound treatment at 40 kHz with chlorine (C40U) rendered the highest bacterial reduction of 3.63-log CFU/g at 5 min. We also observed that a higher log reduction was achieved with ultrasound when combined with C or P compared to that with W. Specifically, there was a significantly greater *S.* Newport reduction on tomato treated by C25/40U and P25/40U compared to those treated with W25/40U. For example, C25U and C40U for 1 min achieved 1.31 and 1.43-log CFU/g more reductions compared to the sample treated with W25U and W40U, respectively. The additional log reduction by P25U and P40U on tomato were 1.21 and 1.83-log CFU/g, respectively. Interestingly, ultrasound at 25 kHz and 40 kHz did not result in a significant difference in *S.* Newport reduction, and neither did longer ultrasound treatment times.

**Figure 2:**
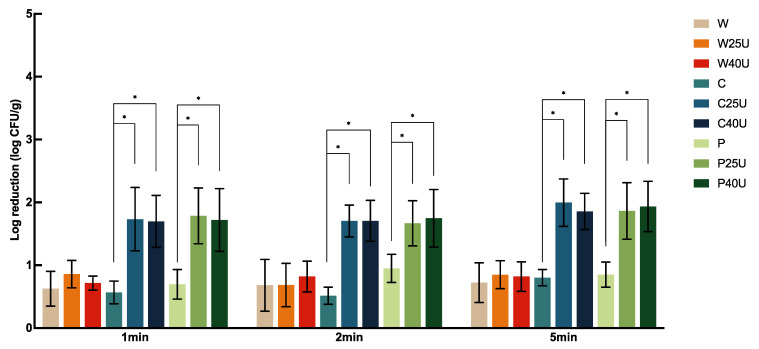
Population dynamics of *Salmonella enterica* Newport 36796 on spinach treated with water (W), water with 25/40 kHz ultrasound (W25/40U), chlorine (C), chlorine with 25/40 kHz ultrasound (C25/40U), peroxyacetic acid (P), or peroxyacetic acid with 25/40 kHz ultrasound (P25/40U) for 1, 2, or 5 min. Data are represented as mean and standard deviation (*n* = 8). Selected statistical analysis results on the figure depict significant differences between population reductions on spinach treated with the same washing solution with and without ultrasound; * indicates significant differences in populations. All other significant differences are indicated in the text.

Figure 2 shows the population changes of *S*. Newport on spinach leaves with ultrasound and sanitizer treatments. There was no significant difference in *S.* Newport reduction between different treatment lengths except for those treated with C. Similar to the results on tomato, the reduction in spinach did not change significantly between the two ultrasound frequencies. W resulted in a reduction of 0.63–0.72-log CFU/g of *S.* Newport, whereas W25/40U only improved the reduction by less than 0.25 log CFU/g. In contrast, C25U, C40U, P25U, and P40U significantly increased the reduction by 1.16, 1.13, 1.09, and 1.02 log CFU/g at 1 min, respectively, similar to those treated at 2 and 5 min. Overall, reductions of 1.67–2.00-log CFU/g on spinach were achieved by simultaneous use of ultrasound and sanitizers, where the highest reduction was achieved by C25U at 5 min.

**Figure 3:**
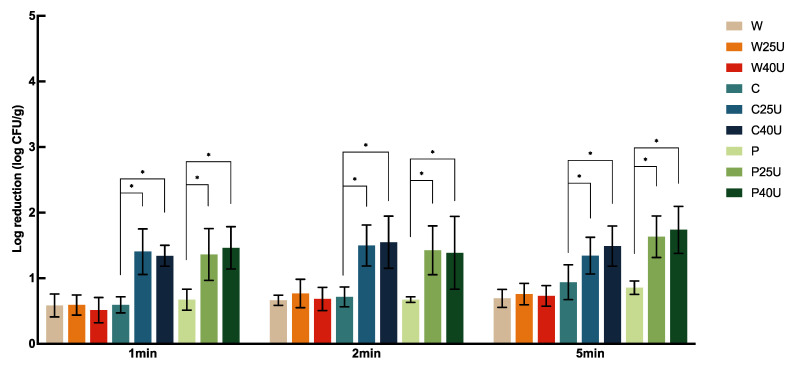
Population dynamics of *Salmonella enterica* Newport 36796 on iceberg lettuce treated with water (W), water with 25/40 kHz ultrasound (W25/40U), chlorine (C), chlorine with 25/40 kHz ultrasound (C25/40U), peroxyacetic acid (P), or peroxyacetic acid with 25/40 kHz ultrasound (P25/40U) for 1, 2, or 5 min. Data are represented as mean and standard deviation (*n* = 8). Selected statistical analysis results on the figure depict significant differences between population reductions on iceberg lettuce treated with the same washing solution with and without ultrasound; * indicates significant differences in populations. All other significant differences are indicated in the text.

Figure 3 illustrates the population dynamics of *S.* Newport on iceberg lettuce with ultrasound and sanitizer treatments. Similar to what we observed on spinach, no significant difference was found in *S.* Newport reduction on iceberg lettuce among the different washing times and ultrasound frequencies. The application of W with U did not increase the population reduction on lettuce significantly. Nevertheless, the combination of ultrasound with both sanitizers did significantly increase the *S*. Newport reduction, when compared to the use of sanitizers alone, by 0.41–0.88 log CFU/g.

The combined use of ultrasound and chemical sanitizers improved the reduction of *S.* Newport on tomato more than on spinach and iceberg lettuce. When treated under the same conditions (e.g., frequency, sanitizer, and length of time), *S*. Newport displayed similar reductions on spinach and iceberg lettuce.

### 3.3. Population Dynamics of L. monocytogenes on Fresh Produce

Grape tomato, spinach, and iceberg lettuce were inoculated with *L. monocytogenes* and were tested with the same treatment combinations (W, W25U, W40U, C, C25U, C40U, P, P25U, and P40U for 1, 2, or 5 min) as with *S*. Newport. The *L. monocytogenes* inoculation level on tomato, spinach, and iceberg lettuce prior to treatment was 7.71 ± 0.75, 8.40 ± 0.22, and 8.40 ± 0.33 log CFU/g, respectively.

**Figure 4:**
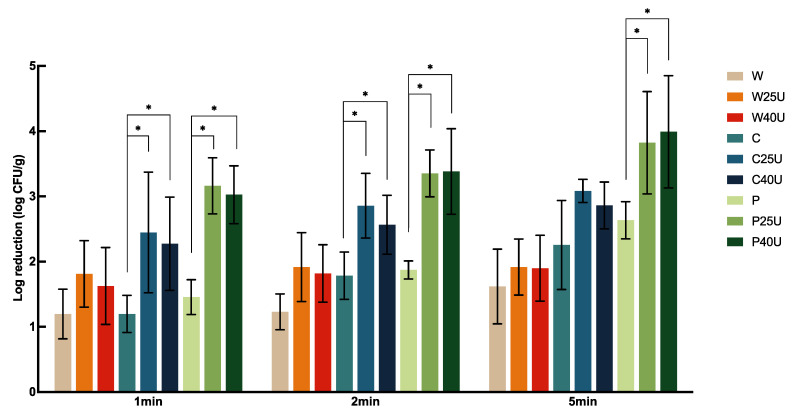
Population dynamics of *Listeria monocytogenes* LS810 on grape tomato treated with water (W), water with 25/40 kHz ultrasound (W25/40U), chlorine (C), chlorine with 25/40 kHz ultrasound (C25/40U), peroxyacetic acid (P), or peroxyacetic acid with 25/40 kHz ultrasound (P25/40U) for 1, 2, or 5 min. Data are represented as mean and standard deviation (*n* = 8). Selected statistical analysis results on the figure depict significant differences between population reductions on grape tomato treated with the same washing solution with and without ultrasound; * indicates significant differences in populations. All other significant differences are indicated in the text.

The population of *L. monocytogenes* on grape tomato treated with different conditions is shown in Figure 4. Treatment of W for 1, 2, and 5 min achieved 1.20 to 1.62 log-CFU/g reduction of *L. monocytogenes* on tomato, but with no significant difference between treatment times. Tomatoes treated with C and P showed significantly higher reductions at 1- or 5-min. However, there was no significant difference between the two sanitizers at the same treatment times. Similar observations were obtained when ultrasound was incorporated. There was no significant difference in the population reduction using 25 or 40 kHz ultrasound. W and U did not render a significantly higher reduction compared to W alone. Ultrasound significantly enhanced *L. monocytogenes* reductions compared to the use of chemical sanitizers alone. Specifically, 1 min ultrasound treatment at 25 kHz with C and P achieved 1.25 and 1.7-log CFU/g more reductions of *L. monocytogenes* compared to the use of sanitizers alone, respectively, doubling the reduction of using ultrasound alone. On average, ultrasound in combination with C or P achieved a 2.27–3.99 log CFU/g reduction of *L. monocytogenes* on tomato; the highest reduction was achieved by P40U at 5 min.

**Figure 5:**
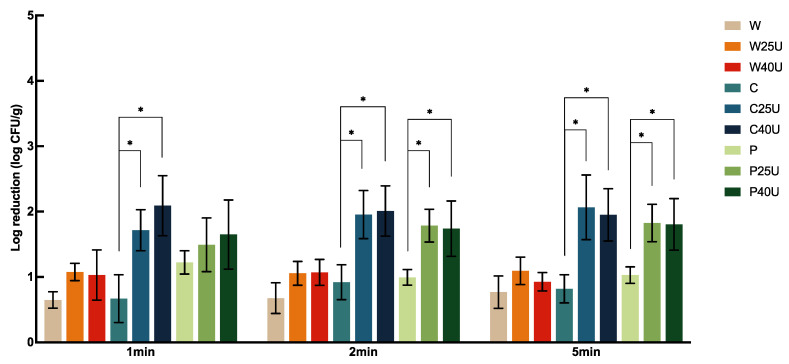
Population dynamics of *Listeria monocytogenes* LS810 on spinach treated with water (W), water with 25/40 kHz ultrasound (W25/40U), chlorine (C), chlorine with 25/40 kHz ultrasound (C25/40U), peroxyacetic acid (P), or peroxyacetic acid with 25/40 kHz ultrasound (P25/40U) for 1, 2, or 5 min. Data are represented as mean and standard deviation (*n* = 8). Selected statistical analysis results on the figure depict significant differences between population reductions on spinach treated with the same washing solution with and without ultrasound; * indicates significant differences in populations. All other significant differences are indicated in the text.

For spinach, the population reduction of *L. monocytogenes* is presented in Figure 5. There was no significant difference in *L. monocytogenes* reduction between different treatment lengths except for the samples treated with P. Similar to the population reduction in tomato, the reduction in spinach did not change significantly between the two ultrasound frequencies. W caused 0.65–0.77 log CFU/g reduction of *L. monocytogenes*, and W25/40U only increased the reduction by less than 0.39 log CFU/g, with no significant difference. On the contrary, C or P combined with ultrasound for 1, 2, or 5 min all significantly increased the reduction of *L. monocytogenes* compared to C or P for the same treatment length. The only exception was found for P25/40U compared to P. C25U, C40U, P25U, and P40U for 2 min significantly increased the reduction by 1.03, 1.09, 0.80, and 0.75 log CFU/g compared to C and P for 2 min, respectively. Similar results were observed on the spinach treated with different conditions for 5 min. Reductions of 1.49–2.09 log CFU/g on spinach were obtained when ultrasound was combined with sanitizers, with the highest reduction being achieved by C40U for 1 min.

**Figure 6:**
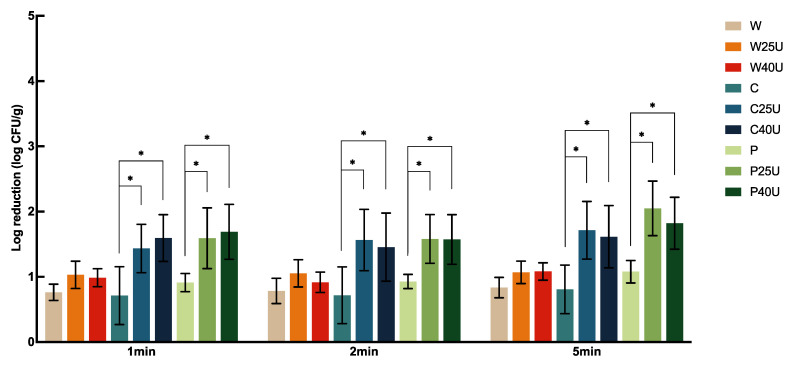
Population dynamics of *Listeria monocytogenes* LS810 on iceberg lettuce treated with water (W), water with 25/40 kHz ultrasound (W25/40U), chlorine (C), chlorine with 25/40 kHz ultrasound (C25/40U), peroxyacetic acid (P), or peroxyacetic acid with 25/40 kHz ultrasound (P25/40U) for 1, 2, or 5 min. Data are represented as mean and standard deviation (*n* = 8). Selected statistical analysis results on the figure depict significant differences between population reductions on iceberg lettuce treated with the same washing solution with and without ultrasound; * indicates significant differences in populations. All other significant differences are indicated in the text.

Figure 6 illustrates the population reduction of *L. monocytogenes* on iceberg lettuce treated with different washing conditions for different lengths of time. Similar to the results for spinach, no significant difference was observed for the reduction of *L. monocytogenes* on iceberg lettuce between different washing times and different frequencies. W with ultrasound did not increase the population reduction on lettuce significantly compared to water treatment alone. However, ultrasound coupled with C or P significantly increased the population reduction compared to sanitizers alone to give a total reduction between 1.44–2.05 log CFU/g.

In summary, ultrasound, in combination with sanitizers, reduced *L. monocytogenes* populations on tomato significantly more than those on spinach and iceberg lettuce. However, there was no significant difference in population reductions on spinach and iceberg lettuce treated with the same frequency, sanitizer, or length of time. There was also no significant difference in the population reduction of the two pathogens on the same fresh produce matrix treated with the same washing condition.

## 4. Discussion

Due to the high incidence of bacterial contamination in minimally processed fresh produce, foodborne pathogens such as *S. enterica*, *L. monocytogenes*, and STEC pose serious safety concerns to public health [21,22,23,24,25,26,27]. Our study evaluated the efficacy of power ultrasound-based hurdle technology on the reduction of pathogens on fresh produce surfaces under different wash conditions. *S.* Newport 36796 and *L. monocytogenes* LS810 were tested in this study to represent a Gram-negative and a Gram-positive bacterial pathogen causing human foodborne outbreaks, respectively. Chlorine is the most commonly used sanitizer in the fresh produce industry; several other alternatives include PAA, lactic acid, and chlorine dioxide [28]. In this study, we focused on the use of chlorine and PAA in combination with power ultrasound.

It is suggested that as low as 25 ppm free chlorine is capable of preventing cross-contamination of pathogens among tomatoes during washing steps [29] and that 100 ppm can eliminate the cross-contamination of tomato even in the presence of high organic load [30]. For PAA, the maximum concentration for washing fresh produce is 80 ppm [30]. One study suggested that 40 ppm of PAA is capable of efficiently reducing the cross-contamination of apples [31]. Based on these studies, we tested 50 ppm chlorine and 40 ppm PAA in this study.

We selected three produce matrices, grape tomato, spinach, and iceberg lettuce, which display some distinctive surface properties, and, more importantly, were associated with previous foodborne disease outbreaks [4]. The treatment lengths, 1, 2, and 5 min, were tested because treatment time beyond 5 min is less practical for industry use. A short washing time not only lowers the cost of but also minimizes possible damage to fresh produce that could otherwise be caused during the extended washing process.

Overall, our study showed that washing with water alone resulted in a 0.50–1.62 log reduction of *S.* Newport and *L. monocytogenes* on grape tomato and a 0.59–0.84 log reduction on spinach and lettuce, which was optimal for reducing pathogens in fresh produce. It has been demonstrated that washing with water for 2 min led to only 0.15 log and 0.10 log reduction after 24 h and 48 h attachment of *L. monocytogenes* on apples [9]. While higher log reductions were achieved in this study, the study by Shen et al. suggested that the attachment time, or the length of drying of the inoculum on the fresh produce surface, has an impact on the efficacy of sanitizer on pathogen reduction. It was speculated that an extended attachment period prior to wash treatment facilitated bacterial attachment and aggregation on the fresh produce [32]. For example, 2 min treatment with 80 ppm PAA showed a significant difference in the reduction of *L. monocytogenes* on apples between 48 h and 24 h of attachment time [9]. The higher reduction rates of pathogens in this study may be a result of the shorter attachment time of *S.* Newport and *L. monocytogenes* on tomato, spinach, and lettuce.

We demonstrated that washing with 40 ppm chlorine could lead to a 1.20 to 2.56 log reduction of pathogens on grape tomato. This is similar to findings by Bolten et al., who observed a 2–3 log reduction of *Salmonella* on grape/cherry tomato washed in a packinghouse dump tank with 25–150 ppm free chlorine but with no significant difference in the *Salmonella* reduction across the different chlorine concentrations [33]. For PAA treatment, a 2 min treatment of 40 ppm PAA reduced the *L. monocytogenes* population on apples by 1.30 log CFU/apple [31]. Lippman et al. demonstrated a 1.30 log reduction of *Salmonella* on shredded lettuce by washing with 40 ppm PAA for 2 min, comparable to what we found on spinach and lettuce in this study [34].

Implementation of hurdle technology for minimally processed fresh produce has great potential to meet the ever-growing market demand for safe and high-quality food. Hurdle technology can improve food safety while preserving food quality through the application of multiple intervention treatments at lower intensities [35]. Here, we evaluated the use of power ultrasound in combination with antimicrobial sanitizers, namely chlorine, and PAA. Our results demonstrated that some additional pathogen reduction of 0.44, 0.75, and 0.63 on grape tomato, spinach, and iceberg lettuce could be achieved when ultrasound and chemical sanitizers were used in combination, compared to the results of using a single technology alone.

Several other studies also reported some synergistic effects of combined ultrasound treatment with other types of chemical sanitizers or antimicrobial compounds [12,13,36,37,38,39]. For example, an additional 0.2 to 0.47 log reduction of *L. monocytogenes*, *S. enterica*, and *E. coli* O157:H7 on lettuce was obtained by the combined 5 min treatment of 40 kHz ultrasound and 2% organic acids compared to using separate treatments [39]. The enhanced antimicrobial effects of ultrasound and sanitizer hurdle treatment can be partially explained by the physical detachment of bacteria from the produce surface due to the shear force generated by ultrasound, as well as the physical–chemical disruption of bacterial cellular structures and functions contributed by both treatments. It was reported that a combination of ultrasound and surfactants, such as Tween-20, can enhance the detachment of bacteria from the surface of fresh produce [12]. For the inactivation of bacteria, one study applied Transmission Electron Microscopy to demonstrate the morphological changes of *L. innocua* after 20 kHz ultrasound treatment alone and combined treatment with citral [13]. The authors observed changes in the cell membranes and intracellular structures after 5 min of the ultrasound treatment. Whereas, with the addition of citral, changes to the cell membrane permeability, leakage of intracellular components, and eventual cell disruption were observed, which suggested that ultrasound treatment accelerated bacterial cell damage [13]. Such damage may facilitate the penetration of antimicrobial chemicals into the bacteria and reach optimal inactivation. Chlorine and PAA are both able to alter cell membrane permeability and oxidize the intracellular substances; ultrasound treatment may play a synergistic role to enhance cell deformation and thus increase the antimicrobial effect.

As to the effect of ultrasound frequency on pathogen reduction, we compared 25 kHz and 40 kHz operated at a similar acoustic power density. The results suggested that there was no significant difference in pathogen reduction between the two frequencies. Similar findings were reported by Zhou et al. [40], where the removal of *E. coli* O157:H7 on spinach by a continuous flow washing system equipped with ultrasound transducers did not change significantly between 25 kHz and 40 kHz, whereas the 75 kHz led to less pathogen reduction. This can be partially explained by the fact that cavitation bubbles formation and collapse under ultrasonication are caused by compression and pulling of the liquid particles. Ultrasonication at lower frequency allows a longer time for bubbles to grow before collapsing due to slower compression and rarefaction, which in turn results in fewer but larger bubbles. On the contrary, ultrasound with higher frequency produces smaller but more bubbles. Higher energy and forces generated by the larger cavitation bubbles than those by small bubbles lead to a stronger bactericidal effect to reduce the pathogen load on fresh produce [41]. Joyce et al. evaluated the reduction of *E. coli* treated with 20, 40, and 580 kHz ultrasound at the same acoustic power density levels and showed no significant difference between 20 and 40 kHz treatments but a significantly lower pathogen reduction with 580 kHz [42].

## 5. Conclusions

In summary, this study demonstrated that the combined use of power ultrasound (at 25 or 40 kHz) and chemical sanitizers enhanced the reduction of pathogens on fresh produce by 1.34 to 3.63 logs depending on the fresh produce matrix. The efficacy of such hurdle treatments was not impacted by different treatment times and low ultrasound frequencies, nor different bacterial strains. However, the surface characteristics of the fresh produce used in this study had a significant impact on the efficacy of ultrasound on pathogen reduction. Synergistic effects were observed with the hurdle treatment of ultrasound and sanitizers, compared to the cumulative effects of the single treatments. As the industry employs washing systems on a much larger scale, it becomes crucial to validate the effectiveness of ultrasound-based hurdle treatment in an industrial setup. The findings of this study suggest that washing fresh produce with 40 kHz ultrasound and 50 ppm chlorine for 1 min is the most efficient approach when considering practicality in the industry. Therefore, the efficacy of ultrasound-based hurdle treatment in a scaled-up continuous-flow washing system will be investigated in the future. Sensory evaluations on the minimally processed produce items will also be incorporated in a future study to determine the potential effects of power ultrasound treatments on fresh produce quality.

## Data Availability

All related data and methods are presented in this paper. Additional inquiries should be addressed to the corresponding authors.
